# Investigating the effect of *Nigella sativa* on the testicular function of first-generation offspring of mice treated with titanium oxide nanoparticles 

**DOI:** 10.22038/AJP.2023.22308

**Published:** 2023

**Authors:** Morteza Abouzaripour, Erfan Daneshi, Fariba Amiri, Sherko Naseri, Azra allahveisi

**Affiliations:** *Cellular and Molecular Research Center, Research Institute for Health Development, Kurdistan University of Medical Sciences, Sanandaj, Iran *

**Keywords:** Nigella Sativa, Sperm, Titanium, Nanoparticles

## Abstract

**Objective:**

Nanoparticles include primary particles with at least one of their dimensions being less than 100 nm. The goal of this research was to determine the possible protective role of *Nigella sativa* (NS) against toxic effects mediated by titanium oxide nanoparticle (TNP).

**Materials and Methods:**

30 adult mice (10 males and 20 females) were used. After mating, the pregnant female mice were randomly divided into 4 study groups (n=5 mice in each group). From the 13th day of gestation until delivery, the mice were given TNP and NS. After delivery, 10 newborn male mice were selected from each group and kept under standard conditions until puberty according to the previous grouping (4 groups). The epididymis of each mouse was removed and the sperm was collected for the evaluation of *in vitro* fertilization and testis for histopathology and spermatogenesis of *in vitro* fertilization of first-generation mice.

**Results:**

No significant difference was observed between the NS group and the control group (p>0.05). In the TNP, a degree of epithelial lysis and a significant decrease in sperm motility was observed (p<0.05) compared with the control group. In the TNP and NS group, NS had an ameliorating effect on TNP-induced testicular germ cell damage (p<0.05).

**Conclusion:**

In the present study, it was found that NS had no destructive effect on the germinal epithelium. However, NS had an ameliorating effect on TNP-induced testicular germ cell damage in mice.

## Introduction

Spermatogenesis is a series of events, leading to the formation of spermatozoids. Many factors can affect spermatogenesis, leading to reduced fertility or infertility (Jiang et al., 2014 ▶). The balance between differentiation, germ cell proliferation, and apoptosis is crucial for controlling spermatogenesis. Altering any of these processes may lead to the onset of testicular diseases (de França et al., 1993 ▶). Due to the complexity of the cellular interactions that occur in the testis, toxic compounds can disrupt sperm production (Krzastek et al., 2020 ▶).

Many experimental studies have demonstrated the negative and destructive effects of nanoparticles on male and female germ cells (Han et al., 2016 ▶). Nanoparticles encompass primary particles with at least one of their dimensions being less than 100 nm (Hasan, 2015 ▶). Previous studies have shown the accumulation of nanoparticles in various tissues and the destruction of the blood-testis and blood-brain barriers (McCarthy et al., 2014 ▶). Among various metal nanomaterials, titanium oxide nanoparticles (TNPs) are used in a variety of consumer products (Abouzaripour et al., 2019 ▶). Today, TNPs are used in the production of all types of paints, cosmetics, sunscreens, clothes, electronics, supplements or additives in food and candy, surface coatings, and many other things (such as window glasses, walls, and asphalt) (Lee et al., 2010 ▶). Previous studies have shown TNP as a cause of disruption in mitosis, DNA damage and oxidative stress) Gurr et al., 2005 ▶). Nanomaterials can accumulate in the mitochondria and nucleus of the cell and cause the formation of reactive oxygen species (ROS), become a gene mutation (Alkilany et al., 2010 ▶). According to a previous study, TNPs can cause harmful effects on the testis and other tissues in mice (Orazizadeh et al., 2014 ▶). *Nigella sativa *(NS) is an annual flowering plant in the family Ranunculaceae. It is native to eastern Europe and western Asia and has several therapeutic effects (Hannan et al., 2021 ▶). NS has an antioxidant function since its main components are p-cymene and thymoquinone. Thymoquinone is the major active principle of the oil of NS and has been shown to exhibit anti-tumor activity against breast, lung, prostrate, liver, colon and pancreatic cancer (Karunamoorthi et al., 2013 ▶; Akram Khan and Afzal, 2016 ▶). Previous studies have shown the protective effect of NS on sperm count, actively moving sperms, and sperm normal morphology( Saied M et al., 2020). It has also been demonstrated that NS improves the levels of follicle stimulating hormone (FSH), luteinizing hormone (LH), and testosterone (Soleimani et al., 2014 ▶; Miri et al., 2020 ▶). 

The aim of this study was to determine the possible protective roles of NS against the toxic effects )high dose 100 mg/kg) mediated by TNPs. Concerning the extensive usage of TNP in industries and its existence in air pollution, the protective effect of NS was investigated on the TNP in the first generation. 

## Materials and Methods

**Essential oil extraction of NS**

In order to extract the essential oil of NS, 100 g of the seeds was ground and immediately distilled by water distillation using a Clevenger apparatus for 3 hr (Tongnuanchan and Benjakul, 2014 ▶). Anhydrous sodium sulfate was used to take the moisture content of the essential oil samples. The collected essential oil was placed in a dark glass bottle and kept at 4°C.

**Essential oil analysis**

*Gas chromatography* analysis of the essential oil was performed on a 6890 series instrument** (**Agilent, Wilmington, DE, USA) equipped with a flame ionization detector. The fused silica capillary column (30 m × 0.25 mm) with 0.25 μm film thickness. The temperature was programmed from 60-245°C at 5°C/min; held for 8 min at 60°C and for 10 min at 250°C. The carrier gas was nitrogen. The injector and detector temperatures were 280°C and 300 °C, respectively. The components of the essential oil were identified by comparing their mass spectra with those stored in the Adams mass spectral-retention index libraries (Tongnuanchan and Benjakul, 2014 ▶). The identified components are shown in (Table 1). 

**Table 1:** Chemical composition of black cumin (*Nigella sativa* L.) essential oil

**No.**	**Compounds**	**RI**	**percentage**
1	α-Thujene	932	8.3
2	α-Pinene	937	7.6
3	Camphene	955	0.1
4	Sabinen	977	1.4
5	β-Pinene	979	2.9
6	β-Myrcene	991	0.1
7	α-Phlandrene	1005	0.1
8	p-Cymene	1030	36.1
9	Limonene	1035	1.1
10	γ-Terpinene	1058	2.7
11	α-Terpinolene	1121	7.99
12	Thymoquinone	1257	14.2
13	Thymol	1292	0.09
14	Carvacrol	1305	1.3
15	(E) -Caryophyllen	1424	0.1

RI: Retention Index

**Animals**

In this study, 30 adult Naval Medical Research Institute mice (10 male and 20 female mice) with the age of 6-8 weeks and a weight of 25-30 g were used. The animals were kept under standard laboratory conditions (12 hr dark and 12 hr light cycle, relative humidity of 50±5%, and temperature of 22±3°C) for at least 1 week before mating. The male and female mice were kept separately before mating.

**Experiment**

In the first step, one adult male mouse and two adult female mice were placed in a cage for mating. The next day, a vaginal plaque was considered the first day of gestation. In the second step, the pregnant female mice were divided into 4 groups and received NS and TNP from the 13th day of pregnancy. The control group received normal saline for 7 days. The NS group was gavaged 5 mg/kg of NS for 7 days. The TNP group was administered with 300 mg/kg of TNP by gavage for 7 days (Hooley et al., 2009 ▶). The TNP+NS group received 300 mg/kg of TNP and 5 mg of NS by gavage for 7 days. 

After delivery, 10 male mice in each group were kept with their mothers until puberty. The adult mice were then sacrificed under anaesthesia with ketamine-xylazine for further experiments. This thesis has ethical code number IR. MUK 96.54

**Histopathology**

The right testis of each animal was fixed in Bouin’s fluid. The testes were embedded in paraffin, sectioned (5 µm), and stained with hematoxylin and eosin (H&E) for histopathology and spermatogenesis. Six slides for each mouse and three fields for each slide were provided to check the following parameters: detachment (separation of cells from the seminiferous tubules), sloughing (separation of the cell mass from the germ cells), and cell vacuole (creation of empty space in the seminiferous tubules). In each field, the number of tubules with vacuole, detachment, and sloughing was divided by the total number of tubules in that field and multiplied by 100 (Oatley et al., 2005 ▶).

**Spermatogenesis**

To evaluate spermatogenesis, the germinal epithelium was assessed using Johnson’s scoring method (Esmaili-Nejad et al., 2015 ▶). Three fields were checked in each of the prepared slides. In each field, 50 seminiferous tubules were examined at a magnification of 40 and 100. Each tubule was given a score of 1-10. Based on this scoring system, the testicular biopsies were evaluated and given a score of 1 to 10. Complete spermatogenesis was given a score of 10.

**Sperm preparation for** ***in vitro*** **fertilization**

After sacrificing 10 male mice in each group through cervical dislocation, their abdomens were opened. Then, the testicles and epididymis of 10 male mice in each group were separated and three oblique cuts were made on the tail of the epididymis and the beginning of the vas deferens. In this way, the sperms were obtained and added to a 150 μl drop of human tubulin fluid (HTF) for 10 min at 37°C under 5% CO_2_. Then, using a hemocytometer and under an optical microscope, the sperm movements (fast, inactive, and slow) were evaluated and analyzed. In addition, for *in vitro* fertilization, sperm capacitation was allowed to proceed in humidified air for 1-2 hr at 37°C and under 5% CO_2_.

**Oocyte collection**

**Figure 1:**
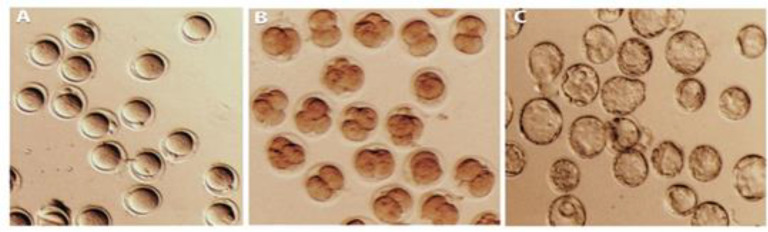
The *in vitro* fertilization and *in vitro* development of mouse embryo. A; the fertilized egg after IVF. B; the 2 and 4 cells after IVF. C; the blastocyst after IVF

The female mice were super-ovulated by intraperitoneal (IP) injection of 5 IU of pregnant mare’s serum gonadotropin (PMSG) (Sigma-Aldrich Germany). After 48 hr, 5 IU of human chorionic gonadotropin (HCG) (Sigma-Aldrich Germany) was injected. The ova were collected 14 hr post-HCG injection. The oviducts were completely removed and placed into 1 ml human tubulin fluid (HTF) at 37°C. The cumulus cells were obtained and then, transferred to 200 µl of HTF medium (Figure 1).

***In vitro*** **fertilization (IVF)**

*In vitro *fertilization was carried out in 200 µl drops of HTF. The sperm was added to 50 µl IVF drops containing cumulus-oocyte complexes in the HTF medium. Then, the mixture was incubated at 37ºC for 6 hr. afterward, the ova were washed three times with M2 medium and finally incubated in 50 μl KSOM^AA^. Fertilization was assessed by recording the number of 2-cell embryos 24 hr after the completion of IVF (Figure 1)*.*

***In vitro*** **development (IVD)**

It was observed under a microscope with a magnification of ×100 that the 2-cell embryos were not fragmented or degenerate. The number of zygotes that developed to the blastocyst stage was calculated in each group.

**Statistical analysis**

The data were compared using one-way analysis of variance (ANOVA). The statistical analysis was performed using the SPSS software (version 20) and p<0.05 was considered significant.

## Results

**Histopathology**

**Figure 2:**
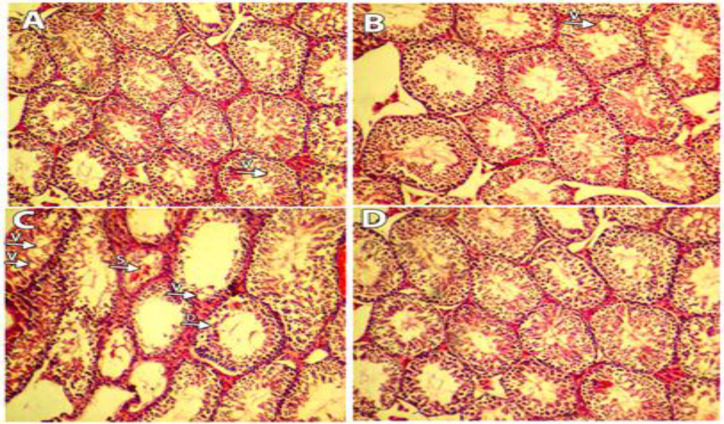
H & E staining of seminiferous tubules. A. Control group: Their mothers received normal saline from the 13th day of pregnancy for 7days. B. NS group: Their mothers received 5 mg/kg NS by gavage from the 13th day of pregnancy for 7days. C. TNP group: Their mothers received 300 mg/kg of TNP by gavage from the 13th day of pregnancy for 7days. D. NS+TNP group: Their mothers received 300 mg/kg TNP and 5 mg NS by gavage from the 13th day of pregnancy for 7days. S: sloughing. V: vacuole. D: detachment

The examination of testicular sections showed normal spermatogenesis in the control group. The normal thickness of the normal spermatogenesis was also seen in the NS group. Also, no significant difference was observed between the control and NS groups regarding the thickness of the germinal epithelium. In the TNP group, there was epithelial lysis, detachment (appearance of breaking off of cohorts of spermatocytes from the seminiferous epithelium), slough (release of clusters of germ cells into the lumen of the seminiferous tubule), vacuole (appearance of empty spaces in the seminiferous tubules), and atrophy of the seminiferous tubules could also be seen. However, in the NS+TNP group, epithelial lysis, detachment, slough, vacuolar, and atrophy of the seminiferous tubules were significantly less than that of the TNP group (p<0.05) (Table 2) (Figure 2).

**Table 2:** Histopathological results in seminiferous tubules in different groups

**Vacuolated**	**Sloughing **	**Detached**	**Normal**	**Percentage tubules **
3.52±.83 3.11±.85 20.52±5.1* 12.48±2.3 #	1.11±.52 2.11±.79 20.55±4.6* 11.46±2.4 #	2.83±1.15 2.66±.88 41.9±5.1* 12.95±2.3 #	92.16±8.3 90.21±9.4 18.42±3.5* 61.42±5.3#	Control NS TNP NS+TNP

Control group: Their mothers received normal saline from the 13th day of pregnancy for 7 days. NS group: Their mothers received 5 mg/kg NS by gavage from the 13th day of pregnancy for 7 days. TNP group: Their mothers received 300 mg/kg of TNP by gavage from the 13th day of pregnancy for 7days. NS+TNP group: Dissimilar letters indicate a significant l difference between different groups. p<0.05 was considered significant. Values are expressed as mean±SD. * and # symbols respectively indicate comparison to the control and TNP groups.

**Spermatogenesis**

**Figure 3:**
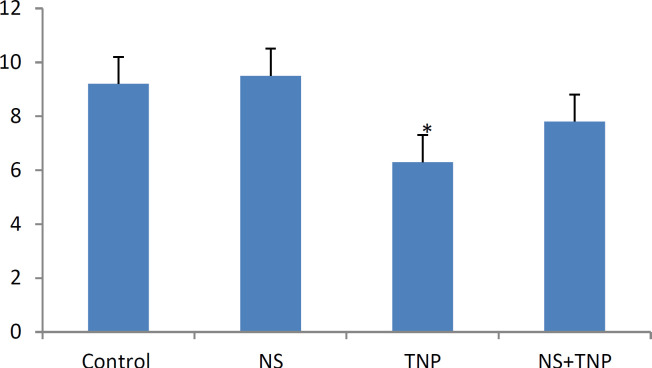
Johnson’s scoring in the control group and different groups. Control group: Their mothers received normal saline from the 13th day of pregnancy for 7days. NS group: Their mothers received 5 mg/kg NS by gavage from the 13th day of pregnancy for 7days. TNP group: Their mothers received 300 mg/kg of TNP by gavage from the 13th day of pregnancy for 7days. NS+TNP group: Their mothers received 300 mg/kg TNP and 5 mg NS by gavage from the 13th day of pregnancy for 7days. *shows the significant difference between the TNP group and the Control group. mean±SD . p<0.01

Spermatogenesis was analyzed in the different groups using Johnson’s technique. Spermatogenesis was normal in the NS group and had no significant difference with that of the control group. In the TNP group, the seminiferous tubules of spermatogenesis were incomplete in some of the sections. In addition, according to Johnson’s score, the score of the TNP group had a significant decrease compared with that of the control group (p<0.01). In the NS+TNP group, fewer tubules had abnormal spermatogenesis compared with the TNP group (p<0.01) (Figure 3).

**Sperm motility**

**Figure 4:**
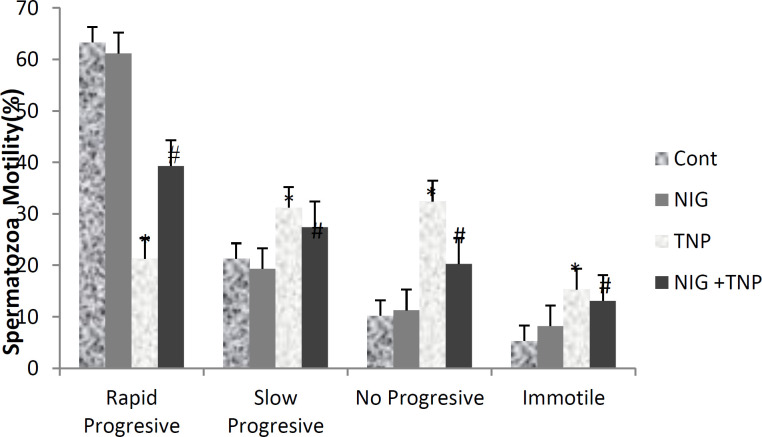
Evaluation of sperm motility in the control and experimental groups. Control group: Their mothers received normal saline from the 13th day of pregnancy for 7days. NS group: Their mothers received 5 mg/kg NS by gavage from the 13th day of pregnancy for 7days. TNP group: Their mothers received 300 mg/kg of TNP by gavage from the 13th day of pregnancy for 7days. NS+TNP group: Their mothers received 300 mg/kg TNP and 5 mg NS by gavage from the 13th day of pregnancy for 7days. Values are expressed as mean±SD (n=10). p<0.05. * and # symbols respectively indicate comparison to the control and TNP groups

In this study, different types of sperm motility were studied. There was no significant difference between the control and NS groups in terms of sperm motility (p<0.01). A significant change in sperm motility was seen in the TNP group compared with the control group. Sperm motility was compensated in the NS+TNP group compared with the TNP group, except that immotile sperm reduced in the NS+TNP group, but no significant compared with the TNP group (Figure 4) (p<0.01).

***In vitro*** **fertilization and development**

The 2-cell stage for IVF and the blastocyst stage for IVD were considered to be positive outcomes. All the collected oocytes were at the M2 stage (mature oocyte). Among the 110 oocytes used for IVF in the control group, 40 reached the 2-cell stage and then 15 of them reached the blastocyst stage. Among the 110 oocytes used for IVF in the NS group, 45 reached the 2-cell stage of which 17 reached the blastocyst stage (p>0.05). Among the 110 oocytes used for IVF in the TNP group, 25 reached the 2-cell stage of which 5 reached the blastocyst stage. The difference between the TNP group and the control and NS groups were significant (p<0.01). Among the 110 oocytes used for IVF in the TNP group, 8 reached the 2-cell stage of which 2 reached the blastocyst stage. The difference between the NS+TNP group and the TNP group was not significant (Figure 1, Table 3).

**Table 3:** IVF and IVD results in in different groups

**Group**	**Number of oocyte**	**Number of two-cells stage**	**Number of blastocysts**
Control	110	40 (36.36%)	15 (13.63%)
NS	110	45 (40.90%)	17 (15.45%)
TNP	110	8 (7.27%) *	2 (1.81%) *
NS+TNP	110	25 (22.72%) #	5 (4.54%) #

Control group: Their mothers received normal saline from the 13th day of pregnancy for 7days. NS group: Their mothers received 5 mg/kg NS by gavage from the 13th day of pregnancy for 7days. TNP group: Their mothers received 300 mg/kg of TNP by gavage from the 13th day of pregnancy for 7days. NS+TNP group: The 2-cell stage for IVF and the blastocyst stage for IVD. Dissimilar letters indicate a significant level between different groups. Values are expressed as mean±SD. * and # symbols respectively indicate comparison to the control and TNP groups.

## Discussion

In recent years, many studies have examined the toxic effects of nanoparticles. However, little attention has been paid to reducing these toxic effects. In this study, the protective effects of NS on spermatogenesis, histopathology, sperm parameters, and *in vitro* fertilization in mice exposed to TNPs were studied. The results of this study showed that TNPs disrupted spermatogenesis and sperm parameters, while NS greatly reduced the toxicity of TNPs. 

The histopathological observations in the present study revealed that TNPs caused morphological changes in the seminiferous tubules. Previous studies have shown that nanoparticles have the ability to cross the blood-testicular barrier and some of them have deleterious and toxic effects on germ cells (Hajshafiha et al., 2013 ▶). Therefore, TNPs can enter the seminiferous tubules by destroying the blood-testicular barrier and directly affect the germ cells or the Sertoli cells. A previous study demonstrated that poly (ethyl methacrylate) nanoparticles passed through biological barriers In another research, the authors showed that nanoparticles impaired the functioning of the genital system of the experimental model. Yamaqishi et al. reported that diesel exhaust nanoparticles cause hypothalamus-pituitary-testis axis disruption and spermatogenesis disruption in rats. They also reported that the production of sex steroids in female rats is also reduced under the influence of diesel exhaust (Yamaqishi, 2011 ▶). They also showed, in another study, that exposing mouse embryos to carbon nanotubes disrupted the reproductive system and caused the testicles to degenerate (Vasyukova et al., 2015 ▶).

In the present study, it was shown that TNPs significantly reduced Johnson’s score, indicating the disruption of spermatogenesis by TNPs. In addition, the results showed that NS effectively increased the Johnson’s score, indicating the beneficial effects of NS on the spermatogenesis process. 

The results of the current study showed that the TNPs reduced the chances of success of IVF and IVD in the mice, whereas NS had no significant effect on the IVF and IVD of the mice. Previous studies have shown the beneficial effects of NS on the reproductive system of rats (Parhizkar et al., 2016 ▶). Histopathological changes such as the loss of the germinal epithelium and the presence of vacuoles in the germinal epithelium were observed in the testicles of mice exposed to TNPs. The occurrence of vacuoles in Sertoli cells showed the degradation of these cells. The separation (sloughing) of premature germ cells from seminiferous tubules by TNPs indicated the effect of this drug on the function of Sertoli cells. According to previous reports, these morphological abnormalities are the symptoms of testicular tissue degradation (Talebi et al., 2013 ▶). It has been shown that when the supportive role of Sertoli cells is undermined, the germinal epithelium degenerates. In a similar study, the effects of N-ethyl-N-nitrosourea on testicular tissue were investigated which showed that it impaired spermatogenesis (Yin et al., 2015 ▶). The separation of cells (sloughing) from the germinal epithelium is probably due to the chemical effects of toxic compounds on the microtubules and cytoskeleton of Sertoli cells (Kopera et al., 2010 ▶). In a previous study, the authors found that titanium dioxide nanoparticles crossed the intestinal epithelial cells by transcytosis (Koeneman et al., 2010 ▶). An ultrastructural examination was not carried out in this study. However, according to the histopathological results, TNPs probably destroyed the blood-testicular barrier by changing the structure of the skeletal system of the Sertoli cells and penetrated the germinal epithelium. The changes in the cytoskeleton of Sertoli cells may weaken their supportive role in the germ cells. *In vivo *and* in vitro *studies have shown that nanoparticles cause histopathological changes in testicular tissue and thus have a significant effect on sperm production and sperm count (Thakur et al., 2014 ▶). By improving the quality and quantity of semen and decreasing lipid peroxidation in rat testicles, NS had a protective effect against testicular damage caused by chlorpyrifos (Rachid et al., 2014 ▶). Therefore, the protective effect of NS may be due to its antioxidant properties. In general, previous studies have shown that NS improves sperm production (Ahmad et al., 2013 ▶). The protective effect of NS may be due to its antioxidant properties (Cho Ping et al., 2014 ▶). 

In the present study, it was found that NS had an ameliorating effect against TNP-induced testicular germ cell damage in mice. NS may be a valuable protective agent to ameliorate spermatogenesis dysfunction and cell loss. Further experiments are needed to clarify the mechanisms of the effect of NS on nanoparticle toxicity.

## Conflicts of interest

The authors have declared that there is no conflict of interest.
